# Differential Requirements for Src-Family Kinases in SYK or ZAP70-Mediated SLP-76 Phosphorylation in Lymphocytes

**DOI:** 10.3389/fimmu.2017.00789

**Published:** 2017-07-07

**Authors:** Frank Fasbender, Maren Claus, Sabine Wingert, Mina Sandusky, Carsten Watzl

**Affiliations:** ^1^Leibniz Research Centre for Working Environment and Human Factors, IfADo, TU-Dortmund, Dortmund, Germany

**Keywords:** natural killer cells, synthetic biology, signal transduction, SLP-76, SYK, Src-family kinases, ZAP70

## Abstract

In a synthetic biology approach using Schneider (S2) cells, we show that SLP-76 is directly phosphorylated at tyrosines Y113 and Y128 by SYK in the presence of ITAM-containing adapters such as CD3ζ, DAP12, or FcεRγ. This phosphorylation was dependent on at least one functional ITAM and a functional SH2 domain within SYK. Inhibition of Src-kinases by inhibitors PP1 and PP2 did not reduce SLP-76 phosphorylation in S2 cells, suggesting an ITAM and SYK dependent, but Src-kinase independent signaling pathway. This direct ITAM/SYK/SLP-76 signaling pathway therefore differs from previously described ITAM signaling. However, the SYK-family kinase ZAP70 required the additional co-expression of the Src-family kinases Fyn or Lck to efficiently phosphorylate SLP-76 in S2 cells. This difference in Src-family kinase dependency of SYK versus ZAP70-mediated ITAM-based signaling was further demonstrated in human lymphocytes. ITAM signaling in ZAP70-expressing T cells was dependent on the activity of Src-family kinases. In contrast, Src-family kinases were partially dispensable for ITAM signaling in SYK-expressing B cells or in natural killer cells, which express SYK and ZAP70. This demonstrates that SYK can signal using a Src-kinase independent ITAM-based signaling pathway, which may be involved in calibrating the threshold for lymphocyte activation.

## Introduction

Natural killer (NK) cells are cytotoxic innate lymphoid cells that are important for early and effective immune responses against cancer and virus-infected cells (1–3), but they can also play a role in tissue homeostasis (4–6). NK cell effector functions are regulated *via* signals from activating and inhibitory surface receptors. Many activating NK cell receptors associate with ITAM-containing partner chains such as DAP12, FcεRγ, or CD3ζ. In humans, activating killer cell Ig-like receptors (KIR-S), CD94/NKG2C-E, and NKp44 associate with DAP12, while NKp46, NKp30, and CD16 pair with CD3ζ and FcεRγ. As T cell and B cell activation relies on ITAM signaling, this pathway has been studied in great detail (7). In a classical view, receptor engagement induces Src-family kinase-dependent phosphorylation of the tyrosines in the ITAM and these in turn form a binding site for the Src-homology domain 2 (SH2) domains of the tyrosine kinases SYK or ZAP70, resulting in downstream signaling. More recent studies on B cell receptor signaling revealed that Syk can phosphorylate and bind to the ITAM tyrosines of Igα and Igβ, which is necessary to open BCR oligomers upon the exposure to multivalent antigens (8). The Src-family kinase Lyn facilitates the opening, but is not absolutely required for this process, whereas it was shown to be necessary to open BCR oligomers upon exposure to monovalent antigens (9).

Natural killer cells express SYK and ZAP70 and ITAM-coupled receptors show defective signaling in mice lacking both of these kinases or lacking DAP12, FcεRγ, and CD3ζ (10). However, NK cells still develop and have cytotoxic activity in these mice as they can also be activated by non-ITAM-based receptors such as NKG2D, 2B4, DNAM-1, and NKp80 (11, 12). It is unclear if and how SYK and ZAP70 cooperate in ITAM signaling in NK cells. While SYK and ZAP70 are very similar in structure, each consisting of two SH2 and a kinase domain, the amino acid sequence of ZAP70 shows less than 50% homology to SYK (13). SYK has been described to be less dependent on Src-family kinases for its catalytic activation, as it is able to trans-autophosphorylate and activate itself (14). In contrast, ZAP70 is highly dependent on Src-family kinases for its activation (15). Other studies have shown that ITAM binding by ZAP70 relieves an auto-inhibitory conformation of ZAP70 and that phosphorylation of specific tyrosine residues within the interdomain separating the C-terminal SH2 domain and the kinase domain by Src-family kinases are important for regulating the activity of ZAP-70 (16, 17).

Most activating receptors need to synergize in order to fully activate resting human NK cells (18, 19). However, exposure to cytokines can modulate NK cell reactivity and enable the activation of NK cells upon triggering of a single activating receptor (20). The molecular basis for this switch in reactivity is only incompletely understood and a recent study suggested that complementary phosphorylation of tyrosine residues 113 and 128 in SLP-76 by different activating receptors was one mechanism how these receptors can synergize to activate resting NK cells (21). SLP-76 was first described in T cells and plays an important role in TCR signaling but SLP-76 is highly expressed in spleen, thymus, and other peripheral blood leukocytes as well. While one earlier study found SLP-76 to be dispensable for NK cell effector functions (22), these findings were contradicted in later studies taking advantage of inducible SLP-76 KO mice (23) and SLP-76 has been shown to be important for NK cell development, cytotoxicity, and IFN-γ production (24, 25).

In synthetic biology, one aim is to rebuild functional minimal signaling systems, which can then be easily manipulated to enhance mechanistic understanding. Schneider (S2) cells, isolated from *Drosophila melanogaster* embryos in 1972 (26), provide a useful platform for this approach. They easily take up large amounts of DNA enhancing co-transfection while maintaining reasonably high transfection efficiency. The evolutionary distant environment should prevent interference of *Drosophila* proteins with the heterologously expressed mammalian signaling system. Previous studies have used the S2 cell system to rebuild BCR signaling pathways. The expression of SYK and the ITAM-containing Igα was sufficient to result in phosphorylation of SLP-65, a SLP-76-related molecule in B cells (27).

Here, we make use of a synthetic biology approach to study the phosphorylation of SLP-76 by ITAM-based signaling pathways. We show that SLP-76 is directly phosphorylated at tyrosines Y113 and Y128 by SYK after activation by an ITAM motif independent of Src-family kinases. In contrast, ZAP70 requires the co-expression of Src-family kinases to phosphorylate SLP-76. Therefore, we identify a distinct ITAM-based signaling pathway for SYK but not ZAP70 using human NK cell-specific signaling proteins. Comparing human lymphocytes that selectively express SYK (B cells), ZAP70 (T cells), or both (NK cells) after stimulation of their respective ITAM-associated receptors demonstrates that SLP-76 phosphorylation in T cells is more sensitive to inhibition of Src-family kinases than in B or NK cells.

## Materials and Methods

### Schneider Cell Culture and Transient Transfection

Schneider S2-cells (Invitrogen) were grown in Schneider’s *Drosophila* medium (Serva) supplemented with 10% FCS at 26°C without CO_2_. Cells were passaged every 2–3 days to maintain exponential growth. S2-cells were transfected using Lipofectamine 2000 (Life Technologies, Inc.). Cells were transfected with 300 ng DNA of each vector and normalized by empty vector to the sample containing the most plasmids. After 24–48 h CuSO_4_ (0.5 mM) was added to the culture for 36–48 h to induce the expression of proteins. For inhibitor experiments, SykIV inhibitor (Calbiochem, 2.5 µM), SYKII inhibitor (2.5 µM Merck), PP1 (Biomol, 10 or 5 µM), PP2 (Biomol, 10 or 5 µM), or DMSO solvent control were added to the cells 1 h prior to harvesting. CuSO_4_-induced GFP^positive^ cells were enriched by cell sorting (BD Jazz). Purity of GFP-positive cells was greater than 80% after enrichment. Receptor expression was analyzed by FACS using the following antibodies: 2B4-APC (C1.7), CRACC-PE (162.1), and NKp80-PE (5D12) all from Biolegend.

### Cloning

For transient co-transfection experiments in *Drosophila* Schneider cells, the expression vector pRmHa-3 containing an inducible metallothionein promoter (28) was used. The cDNAs of SLP-76, 2B4, CRACC, Lck, Csk, FcεR1γ, DAP10, DAP12, and NKp80 were isolated from human NK cells *via* RT-PCR, inserted into pRmHa-3 and verified by sequencing. The vectors pD-Syk, pD-Zap70, pD-Fyn, pD-SHP-1, pD-TCRZeta, pD-ZetaY1-4F, pD-ZetaY1-6F, and pD-Syk mutants were obtained from BIOSS (Freiburg, Germany) and verified by sequencing. The pD vectors are based on pRmHa-3 using the same promoter and all sequences are of human origin.

### Western Blotting

Equal cell numbers of enriched GFP^+^ S2-cells were lysed on ice in lysis buffer [0.5% Triton X-100, 2 mM EDTA, 10 mM NaF, 20 mM Tris–HCl, 150 mM NaCl, 10% (v/v) glycerol, pH 7.3] supplemented with proteinase and phosphatase inhibitor cocktails (Roche). Cell lysates were separated by reducing SDS-PAGE and detected by Western Blot using the following antibodies: pY113-SLP-76 (J80-373), pY128-SLP-76 (J141-668.36.58) (BD Biosciences); isotype control mouse IgG1 (MOPC-21) (Sigma); SLP-76 (#4958), ZAP70 (D1C10E) (Cell signaling); CD3-Zeta, Syk (SYK-01) (Biolegend); p-CD3ζ (C415.9A, Santa Cruz); Fyn, DAP10, DAP12, FcεRγ, 2B4, SHP-1, GAPDH (GA1R) (Thermo Scientific); and Csk (C14520 Transduction Laboratories). Band intensities corresponding to SLP-76 phosphorylated at tyrosine 128 relative to that of total SLP-76 were quantified using ImageJ software.

### NK Cell Culture and Stimulation

Peripheral blood mononuclear cells (PBMCs) were isolated from blood of healthy donors by Ficoll density gradient centrifugation (PAN-Biotech, Germany). Human NK cells were purified from PBMCs using the Dynabeads Untouched Human NK Cell kit (Thermo Fisher Scientific) according to manufacturer’s instructions. For NK cell activation and expansion, purified NK cells were cultured in 96-well round-bottom plates (Nunc) with irradiated K562-mbIL15-41BBL (kind gift from Dario Campana) in IMDM Glutamax supplemented with 10% FCS and 1% penicillin/streptomycin, IL-2 (100 U/ml, NIH Cytokine Repository) and IL-15 (5 ng/ml, PAN-Biotech). IL-21 (100 ng/ml, Miltenyi Biotec) was added at the first day. NK cells were between 90 and 99% CD3^−^, CD56^+^, and NKp46^+^ as assessed by flow cytometry.

For inhibitor experiments, NK cells were preincubated with the indicated inhibitors or solvent control for 30 min at 37°C and subsequent steps conducted in the presence of inhibitors. For antibody-mediated crosslinking, NK cells were preincubated with the following antibodies at 10 µg/ml for 30 min on ice: NKp80 (5D12), NKp46 (9E2), and CD16 (3G8) from Biolegend. NKp30 (p30-15) and NKp44 (p44-8) were produced in our lab. After washing with medium, NK cells were stimulated by crosslinking with goat anti-mouse F(ab′)_2_ Ab (20 µg/ml, Dianova) at 37°C for 2 min, washed in ice-cold PBS, lysed, and analyzed by Western Blotting as described above.

### Phosflow Analysis

Peripheral blood mononuclear cells were incubated with antibodies against CD3 (UCHT1) and CD28 (CD28.2) (all from Biolegend) to stimulate T cells, CD16 (3G8) (Biolegend) to stimulate NK cells, or F(ab′)2 anti-human IgM + IgG (eBioscience) to stimulate B cells in the presence of DMSO or inhibitors for 30 min on ice. Cells were washed and incubated with secondary Goat anti-mouse antibodies for 15 min, followed by stimulation for 2 min at 37°C. Cells were then fixed by BD Phosflow Fixation buffer and stained for extracellular markers with CD19-AF700 (HIB19), CD56-BV510 (NCAM16.2) and CD3-PerCP (UCHT1) (all from Biolegend). Cells were then permeabilized by BD Perm 2, stained with p-SLP-76 Y128 AF647 (J141-668.36.58) or p-SLP-65 Y84 AF647 (J117-1278) (both from BD) and analyzed by flow cytometry on a BD LSRFortessa.

## Results

### Co-expression of Heterologous Proteins in S2 Cells

**Figure 1:**
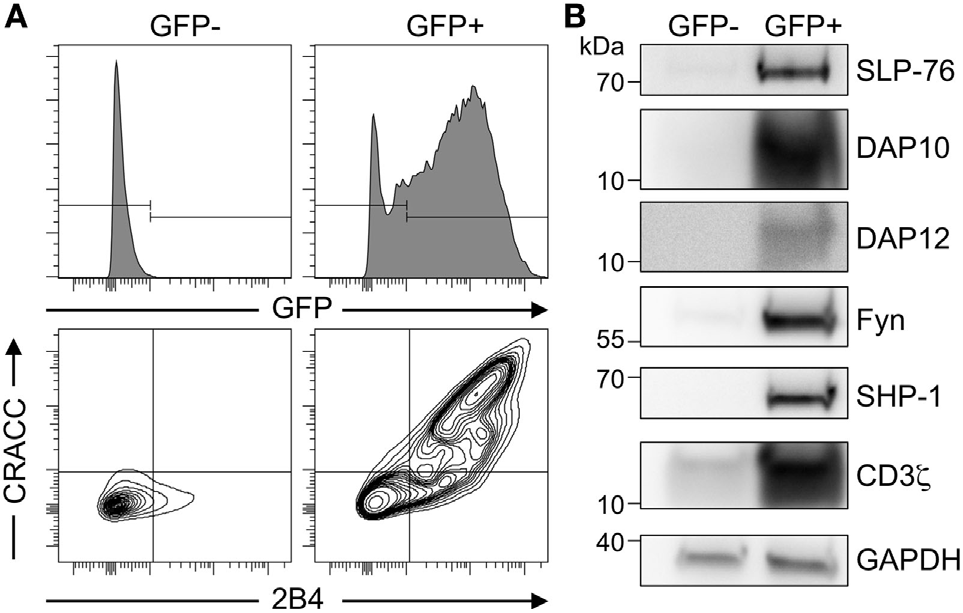
High co-transfection rate of multiple genes in transiently transfected Schneider cells. **(A)** Schneider S2-cells were co-transfected with plasmids coding for 2B4, CRACC, SLP-76, DAP10, DAP12, Fyn, SHP-1, CD3ζ, and GFP. Expression was induced by CuSO_4_. After 36–48 h GFP^−^ and GFP^+^ cells were enriched by cell sorting and analyzed by FACS for expression of GFP, 2B4, and CRACC. Results are representative of three independent experiments. **(B)** Western blot analysis of co-expressed proteins in the total cellular lysate of transiently transfected S2 cells from **(A)** enriched for GFP^−^ and GFP^+^ by cell sorting. GAPDH was used as a loading control. Results are representative of five independent experiments.

To test for the high co-transfection efficiency in S2 cells, we transiently transfected them with plasmids encoding for GFP, SLP-76, DAP10, DAP12, Fyn, SHP-1, CD3ζ, and for the receptors 2B4 and CRACC. This resulted in about 30% GFP-positive cells, which were then sorted by FACS as GFP^positive^ and GFP^negative^. Analysis of protein expression by western blot and flow cytometry clearly indicated that only GFP^positive^ cells expressed high levels of all the transfected proteins while there were almost undetectable levels of the transfected proteins in GFP^negative^ cells (Figure 1). FACS analysis of 2B4 and CRACC confirmed surface expression of both receptors and revealed that most cells co-expressed both receptors. This demonstrates that transfected S2-cells take up all available plasmids and subsequently co-express many heterologous proteins with a reasonably high efficiency. Furthermore, using GFP as a marker, we were able to enrich the co-transfected cells. Thus, the S2-cell system provides a suitable platform for the setup of an artificial signaling system that is easy to manipulate as already demonstrated by others (27, 29–31).

### A Minimal ITAM–SYK–SLP-76 Signaling Pathway

**Figure 2:**
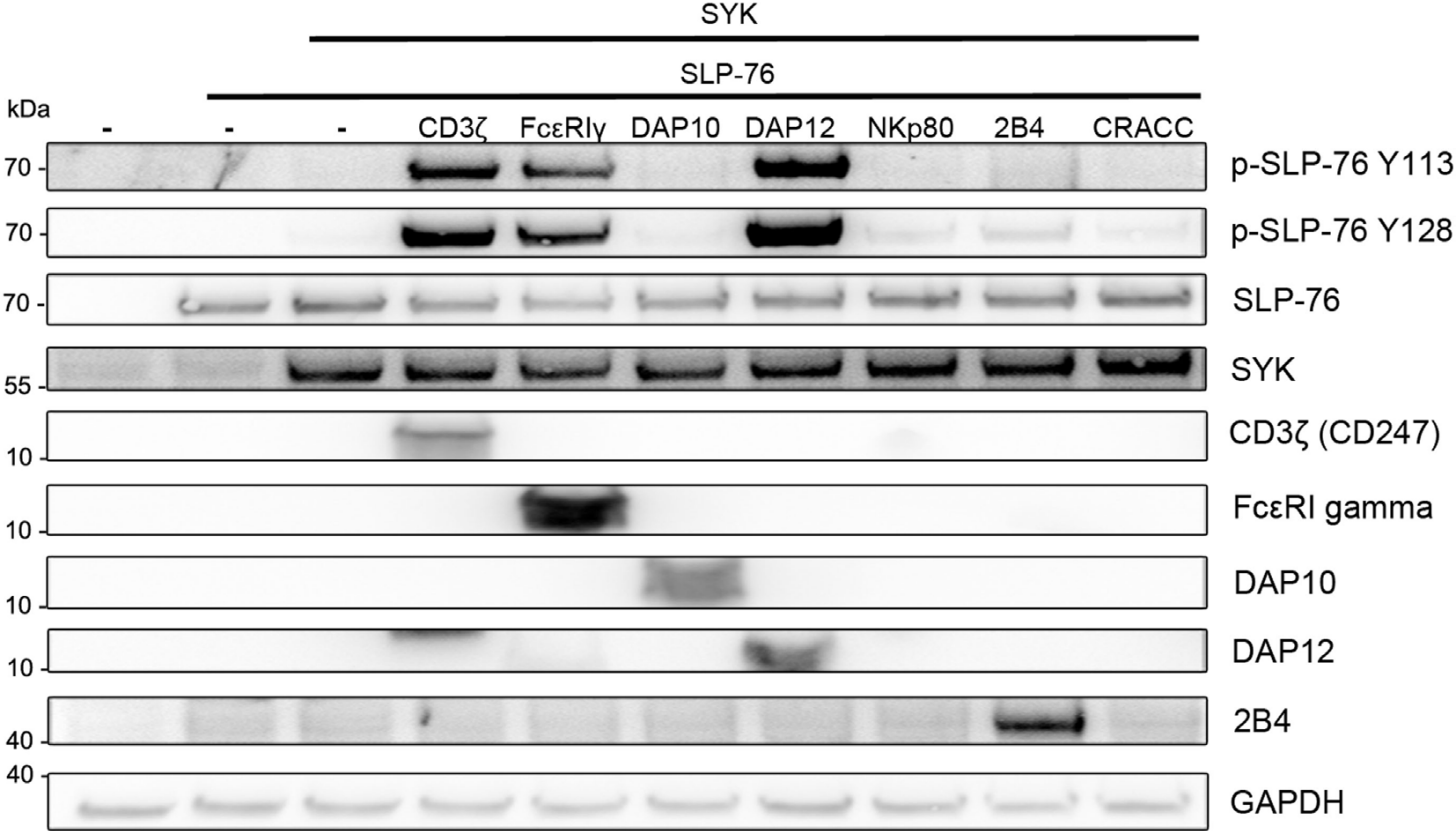
SLP-76 phosphorylation in the presence of SYK and ITAM-containing receptors. S2-cells were co-transfected with the indicated proteins and GFP as a transfection marker. Equal numbers of FACS-enriched GFP^+^ S2-cells were lysed and analyzed by western blot for SLP-76 phosphorylation and the expression of the transfected proteins. Expression of NKp80 and CRACC was verified by FACS analysis (not shown). Results are representative of at least five independent experiments.

We then used the S2-cell system to rebuild an ITAM-based signaling pathway resulting in SLP-76 phosphorylation. Expression of SLP-76 alone in S2-cells did not result in its phosphorylation, demonstrating that *Drosophila* kinases do not significantly phosphorylate human SLP-76 (Figure 2). Co-expression of SYK also did not result in SLP-76 phosphorylation. However, co-expression of SLP-76 and SYK together with ITAM-bearing signaling receptors such as CD3ζ, FcεRγ, or DAP12 induced phosphorylation of SLP-76 at tyrosines Y113 and Y128. NKp80 was shown to stimulate SYK phosphorylation and SYK-dependent cytotoxicity *via* a hemi-ITAM motif (32). However, co-expression of NKp80 did not result in substantial SYK-mediated SLP-76 phosphorylation in the S2-cells (Figure 2). Similarly, co-expression of the NKG2D-associated partner chain DAP10 or the ITSM-containing receptors 2B4 and CRACC also showed very little effect on SYK-mediated SLP-76 phosphorylation compared to the co-expression of ITAM-containing adapter chains. These data establish a minimal signaling pathway from ITAM *via* SYK to SLP-76 without the need for other adapters or kinases.

**Figure 3:**
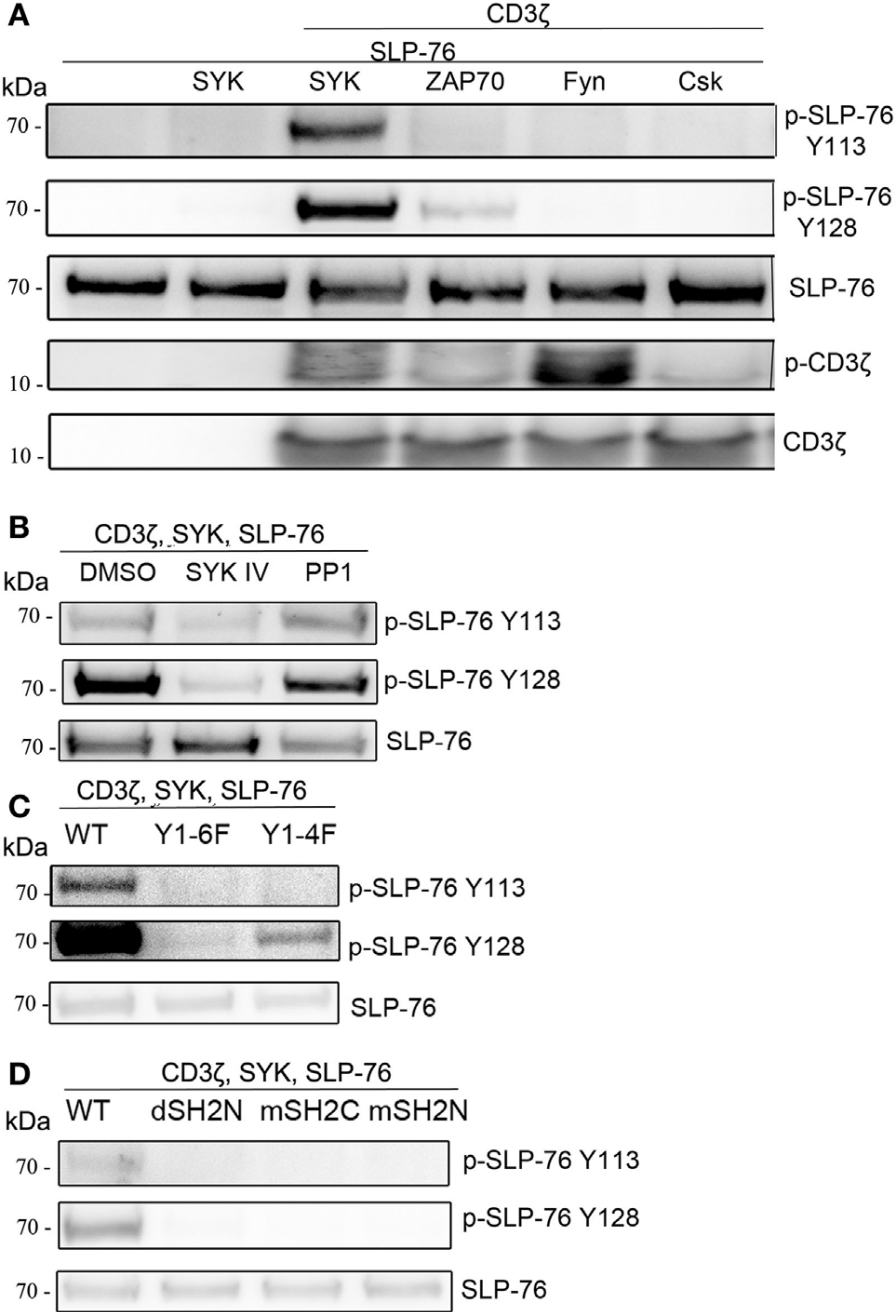
ITAM and SYK-dependent, but Src-independent SLP-76 phosphorylation. S2-cells were co-transfected with the indicated proteins. Equal numbers of FACS-enriched GFP^+^ cells were analyzed by western blot. **(A)** Analysis of SLP-76 and CD3ζ phosphorylation upon co-expression with SYK, ZAP70, Fyn, or Csk. **(B)** S2-cells were co-transfected with GFP, SLP-76, CD3ζ, and SYK. SLP-76 phosphorylation was analyzed in GFP^+^ cells upon treatment with the SYK-family kinase inhibitor SKY IV, the Src-family kinase inhibitor PP1 or DMSO as a carrier control. **(C)** Analysis of SLP-76 phosphorylation upon co-expression of GFP, SLP-76, SYK, and either wild-type (wt) CD3ζ or the indicated CD3ζ ITAM-mutants. **(D)** Analysis of SLP-76 phosphorylation upon co-expression of GFP, SLP-76, CD3ζ and either wild-type (wt) SYK or the indicated SYK SH2 domain mutants. All western blots are representative of at least three independent experiments.

Next, we tested the ability of other kinases to phosphorylate SLP-76 when co-transfected with an ITAM-containing protein. The SYK-family kinase ZAP70 was able to phosphorylate SLP-76 to a lesser extend. While the Src-family kinases Fyn or Lck (not shown) clearly induced CD3ζ phosphorylation, no phosphorylation of SLP-76 was detectable. Also the kinase Csk did not induce SLP76 phosphorylation when co-transfected (Figure 3A). While the typical ITAM signaling pathway requires Src-kinases to phosphorylate ITAM motifs for interaction with the SH2 domains of SYK, in our transfected S2-cells SLP-76 phosphorylation by ITAM-activated SYK was independent of Src-family kinases. This may be explained by the ability of SYK to directly phosphorylate ITAM motifs (27). In line with this, we detected CD3ζ phosphorylation in the presence of SYK and lower phosphorylation in the presence of ZAP70 (Figure 3A).

To further explore the involvement of SYK and Src-family kinases, we used pharmacological inhibitors during the co-expression of CD3ζ, SYK, and SLP-76. Blocking SYK by the inhibitor SYK IV completely blocked the SLP-76 phosphorylation (Figure 3B), demonstrating the kinase activity of SYK is essential for this minimal signaling pathway. In contrast, blocking Src-family kinases with the inhibitor PP1 did not impact SLP-76 phosphorylation, excluding the possibility that *Drosophila* Src-related kinases may be involved.

Using CD3ζ mutants we observed no SLP-76 phosphorylation when all tyrosines of the ITAMs were replaced by phenylalanine (Y1-6F), whereas the presence of a single intact ITAM (Y1-4F) was sufficient to result in some SLP-76 phosphorylation (Figure 3C). Similarly, using SYK mutants, we found that SLP-76 phosphorylation was dependent on both SH2 domains, as we did not detect any phosphorylation when the N-terminal SH2 domain of SYK was deleted (dSH2N) or the N-terminal or the C-terminal SH2 domain was inactivated by mutations (mSH2C, mSH2N) (Figure 3D). These data suggest that the minimal signaling pathway in S2-cells still relies on the interaction of the SYK SH2 domains with the ITAM motif of CD3ζ, despite being independent of Src-family kinases.

### Differential Requirements for Src-Family Kinases

**Figure 4:**
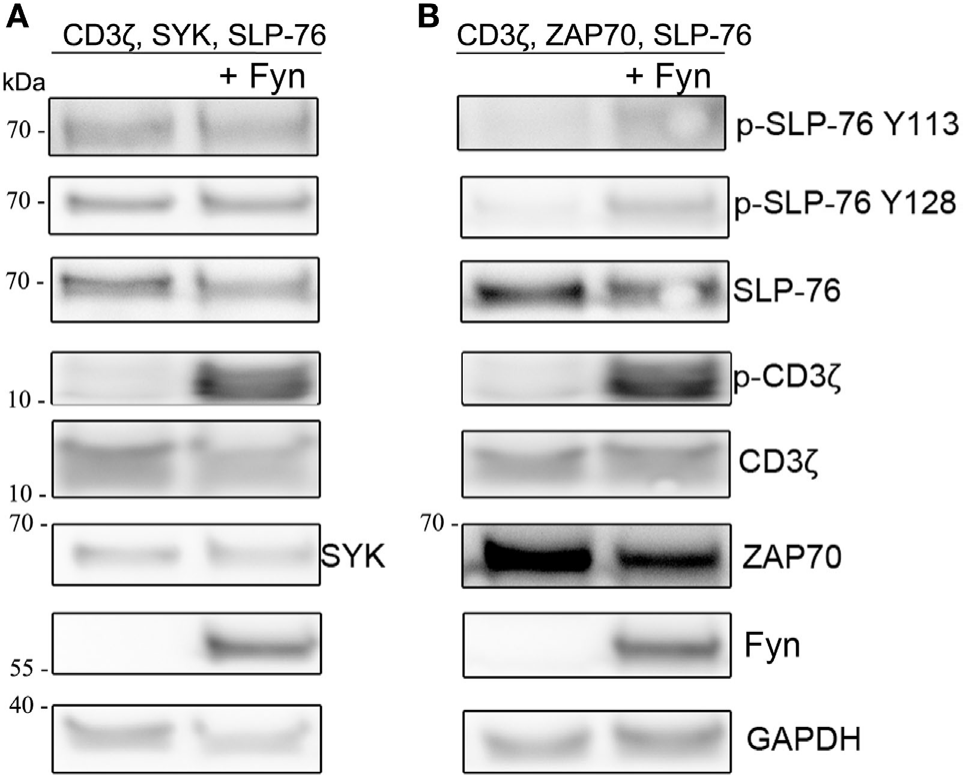
Co-expression of Fyn enhances ZAP70 activity. S2 cells were co-transfected with GFP, SLP-76, CD3ζ, and **(A)** SYK or **(B)** ZAP70 in the absence (left lanes) or presence (right lanes) of Fyn. Equal numbers of FACS-enriched GFP^+^ S2-cells were lysed and analyzed by western blot for SLP-76 and CD3ζ phosphorylation and expression of the transfected proteins. All western blots are representative of at least three independent experiments.

Next we wanted to investigate if the presence of Src-familykinases can enhance SYK or ZAP70-mediated SLP-76 phosphorylation in our system. Co-transfection of Fyn together with CD3ζ, SYK, and SLP-76 did not result in any stronger SLP-76 phosphorylation compared to the absence of Fyn (Figure 4A). This was despite the fact that the presence of Fyn resulted in stronger CD3ζ phosphorylation. In contrast, ZAP70-induced SLP-76 phosphorylation was enhanced when we co-expressed Fyn (Figure 4B). This suggests that SYK can induce SLP-76 phosphorylation *via* a direct ITAM-based signaling pathway independent of Src-family kinases, whereas ZAP70-induced SLP-76 phosphorylation is more dependent on the activity of Src-family kinases.

**Figure 5:**
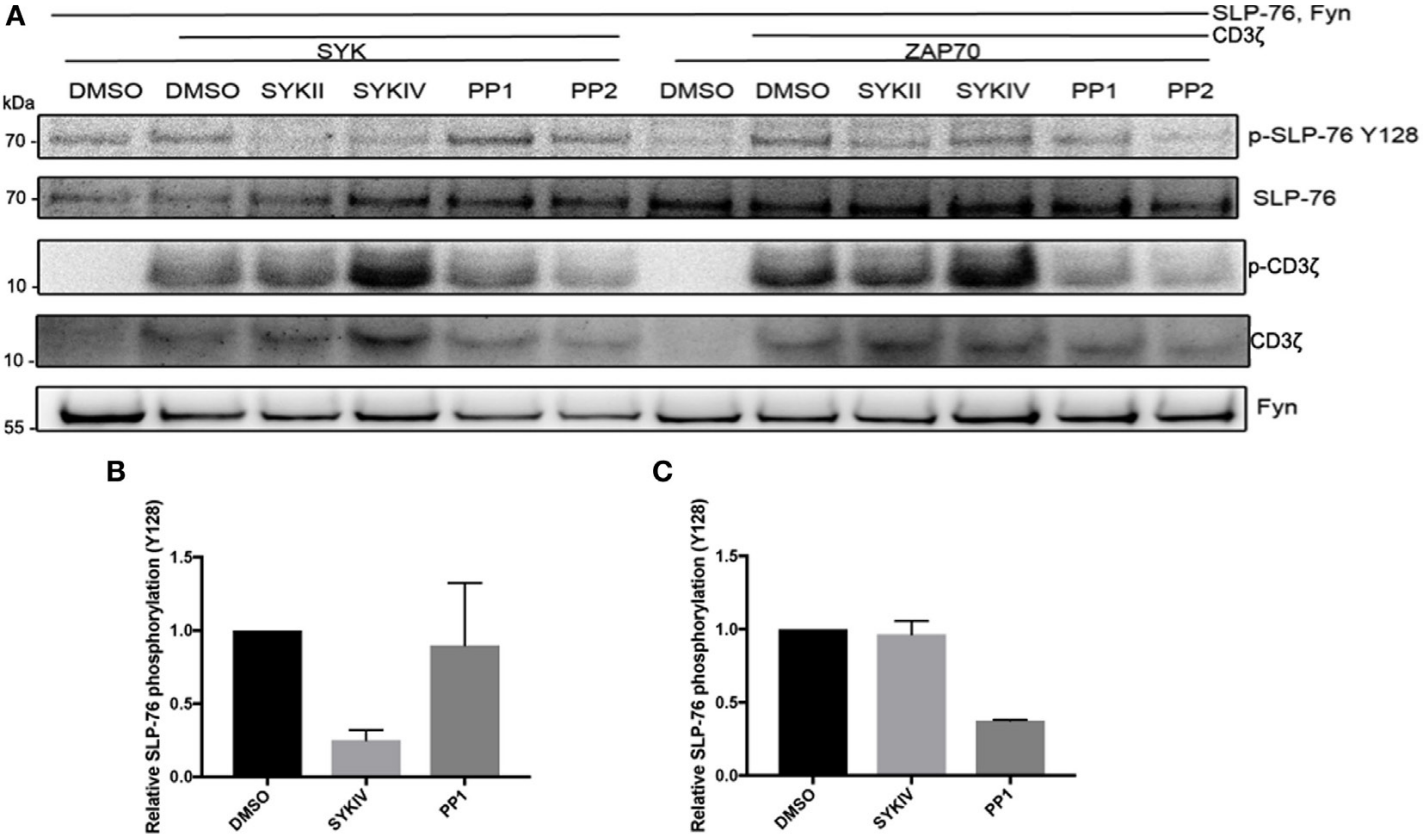
ZAP70 requires co-expression of an ITAM and Src-family kinases activity for activation. **(A)** S2 cells were co-transfected with the indicated proteins and pretreated with SykIV inhibitor (2.5 µM), SYKII inhibitor (2.5 µM), PP1 (10 µM), PP2 (10 µM), or DMSO solvent control 1 h before harvesting. Equal numbers of FACS-enriched GFP^+^ cells were lysed and analyzed by western blot for SLP-76 phosphorylation and expression of transfected proteins. All western blots are representative of at least three independent experiments. **(B)** Quantification of SLP-76 Y128 phosphorylation relative to total SLP-76 expression upon co-expression of CD3ζ, Fyn, SYK, and SLP-76 after pretreatment with the indicated inhibitors or DMSO control. **(C)** Quantification of SLP-76 Y128 phosphorylation relative to total SLP-76 expression upon co-expression of CD3ζ, Fyn, ZAP70, and SLP-76 after pretreatment with the indicated inhibitors or DMSO control.

To further investigate the differential roles of Src-family kinases in SYK and ZAP70 activation, we co-expressed Fyn together with SYK or ZAP70, CD3ζ, and SLP-76 and used pharmacological inhibitors to block the different kinases (Figure 5). We observed that Fyn could directly activate SYK and substitute for its ITAM-mediated activation. In contrast, ZAP70 relied on both Fyn and CD3ζ for its activation as evident by SLP-76 phosphorylation levels. Pharmacological inhibition of SYK clearly inhibited SLP-76 phosphorylation in SYK, Fyn and CD3ζ expressing cells, demonstrating that SYK activity is essential for this signaling pathway and confirming that Fyn is unable to directly phosphorylate SLP-76. In ZAP70 expressing cells, the SYK inhibitors only showed a minor effect on SLP-76 phosphorylation. This may be explained by the fact that the inhibitors are more specific and effective against SYK than against ZAP70. Inhibiting Src-family kinases showed the opposite effect. SYK-mediated SLP-76 phosphorylation was not affected by the inhibition of Src-family kinases, demonstrating again the existence of a Src-family kinase independent ITAM-based signaling pathway. In contrast, ZAP70-mediated SLP-76 phosphorylation was reduced in the presence of Src-family kinase inhibitors. This shows that ZAP70-mediated SLP-76 phosphorylation follows the typical ITAM signaling pathway and relies on the activity of Src-family kinases. In contrast, SYK can operate independent of Src-family kinases and directly lead to SLP-76 phosphorylation in a non-typical ITAM-based signaling pathway.

### A Minimal ITAM Signaling Pathway in SYK-Expressing Lymphocytes

**Figure 6:**
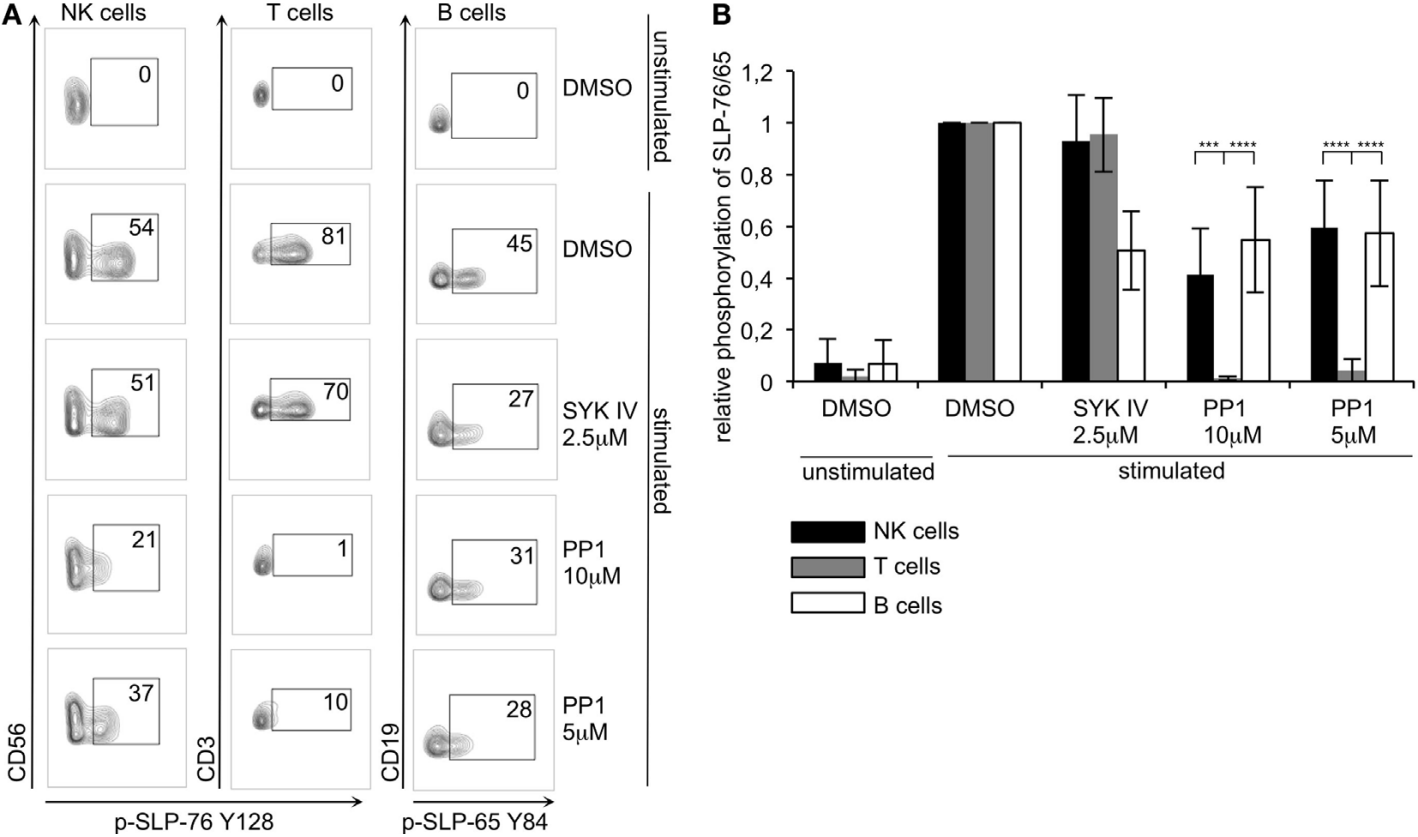
ZAP70 expressing human T cells are more sensitive to inhibition of Src-family kinases than SYK/ZAP70 expressing natural killer (NK) cells or SYK-expressing B cells **(A)** Human peripheral blood mononuclear cells were preincubated in the presence of the indicated inhibitors or DMSO control with mAbs specific for CD16 (to stimulate NK cells), CD3 and CD28 (to stimulate T cells), IgM (to stimulate B cells), or an isotype control (unstimulated) for 30 min on ice, followed by cross-linking with secondary goat F(ab′)_2_ anti-mouse IgG at 37°C for 2 min. Phosphorylation of SLP-76 in NK (CD3^−^CD56^+^) and T (CD3^+^CD56^−^) cells or of SLP-65 in B (CD3^−^CD19^+^) cells was analyzed by flow cytometry using phosphorylation specific antibodies. **(B)** Quantification of (A). Normalized data were subjected to two-way ANOVA to discern significant differences (α = 0,05). Statistical analysis was carried out by using PRISM software (*N* = 5 for NK and T cells; *N* = 3 for B cells). *P*-values: ***<0.001; ****<0.0001.

To test if the minimal ITAM-based signaling pathway that we rebuilt in S2 cells is also operational in human lymphocytes, we first tested for SLP-76 phosphorylation in human NK cells. In IL-2 activated, cultured human NK cells, we observed phosphorylation of SLP-76 at both tyrosines (Y113 and Y128) after stimulating CD16, NKp30, NKp44, or NKp46, all of which are associated with ITAM-bearing adapter chains (Figure S1 in Supplementary Material). In human NK cells, NKp44 associates with DAP12 but is only expressed on activated NK cells (33), while NKp46, NKp30, and CD16 pair with CD3ζ and FcεRγ (34–36). Stimulation of NKp80, which signals *via* a hemi-ITAM, did not result in detectable SLP-76 phosphorylation, whereas the ITSM-containing 2B4 receptor could induce strong SLP-76 phosphorylation at both tyrosines. Additionally, we also observed SLP-76 phosphorylation upon stimulation of the ITAM-associated receptors CD16, NKp30, and NKp46 in freshly isolated, resting human NK cells (data not shown). NK cells express SYK and ZAP70, whereas T lymphocytes only express ZAP70 and B lymphocytes only express SYK. To investigate and compare ITAM-based signaling pathways in these cells, we isolated PBMCs and stimulated NK cells *via* cross-linking CD16 [as this receptor was shown to be efficient in stimulating resting NK cells (18)], T cells *via* crosslinking of CD3 and CD28 and B cells *via* crosslinking of surface IgM in the same sample (Figure S2 in Supplementary Material). We then analyzed SLP-76 phosphorylation in T and NK cells and phosphorylation of the SLP-76 homolog SLP-65 in B cells by intracellular staining and flow cytometry. The stimulation of ITAM-based receptors resulted in SLP-76 phosphorylation in NK cells and T cells and in SLP-65 phosphorylation in B cells (Figure 6). In the presence of the SYK inhibitor SYK IV, the SLP-65 phosphorylation in B cells was reduced, whereas the SLP-76 phosphorylation in T cells was not significantly affected. This mirrors our results of the S2 cells where we saw that the SYK IV inhibitor is more specific for SYK than for ZAP-70. Interestingly, the SYK IV inhibitor also did not significantly affect the SLP-76 phosphorylation in NK cells, suggesting that ZAP-70 can compensate upon inhibition of SYK in these cells. Next we wanted to test the role of Src-family kinases by inhibiting their activity using PP1 in two different concentrations. Both concentrations completely inhibited SLP-76 phosphorylation in T cells, demonstrating that these cells rely completely on the typical ITAM-based signaling pathway. This is in line with our results from the S2 system, where ZAP70-mediated SLP-76 phosphorylation was dependent on the activity of Src-family kinases. In contrast to T cells, we still observed a significant phosphorylation of SLP-65 in B cells and of SLP-76 in NK cells upon inhibition of Src-family kinases. As we stimulated all lymphocytes in the same sample, this experimental setup allowed for a direct comparison of sensitivities to inhibition of Src-family kinases and SYK. This suggests that in SYK expressing B and NK cells a non-typical ITAM-based signaling pathway is operational that is at least partially independent of Src-family kinases. This provides evidence for differential regulation of SYK and ZAP70 by Src-family kinases in primary human lymphocytes.

## Discussion

In our synthetic biology approach, we found SLP-76 phosphorylation upon co-expression of an ITAM-containing receptor, SYK, and SLP-76 in S2-cells. Similar to previous studies investigating the B cell homolog SLP-65 with the same approach (27), the initiation of this signaling pathway did not require engagement of a surface receptor. This may be due to an overexpression of the ITAM-containing signaling chain in the S2-cells or due to the fact that inhibitory proteins, which shield the ITAM sequence from interacting with kinases in resting hematopoietic cells, are missing in the S2-cells. While we only saw SLP-76 phosphorylation upon co-expression of ITAM-containing receptors in the S2-cells, this does not exclude the phosphorylation of SLP-76 upon triggering of non-ITAM activating receptors as other adapters and kinases might build a different signaling pathway. SLP-76 phosphorylation can also be induced by cross-linking of non-ITAM receptors such as NKG2D, 2B4, and DNAM1 [(21); Figure S1 in Supplementary Material]. This indicates that these pathways might function independent of SYK kinase. 2B4 signals *via* an ITSM-motif which is phosphorylated by Src-family kinases, but does not recruit SYK-family kinases (37). While 2B4-triggering in resting human NK cells only induces the phosphorylation of SLP-76 at Y113 (21), we saw 2B4-induced SLP-76 phosphorylation at Y113 and Y128 in IL-2 activated, cultured NK cells, in line with the fact that these activated cells do not rely on co-activating receptors to induce their activity (18). Similarly, NKG2D-DAP10 seems to rely on Src-family kinases (38) and triggers human NK cell cytotoxicity *via* a SYK-independent pathway (39).

Triggering of ITAM-based receptors such as CD16 in NK cells induces the phosphorylation and activation of SYK and ZAP70 (40, 41), and these kinases are essential for the function of the receptors (42). In our S2-cell system, SYK-induced SLP-76 phosphorylation did not require the activity of Src-family kinases. This differs from the typical ITAM-based signaling pathway, where Src-family kinases are required for ITAM signaling. However, as we did not test all Src-family kinases in our S2 system, we cannot exclude the possibility that some Src-family kinases may enhance SYK-mediated SLP-76 phosphorylation.

Our mutagenesis data show that the minimal signaling pathway in S2-cells still relied on the presence of tyrosines in the ITAM receptor and on a functional SH2 domain in SYK. This indicates that this non-typical signaling pathway is still dependent on the interaction of the SYK SH2 domains with the tyrosines of the ITAM, despite being independent of Src-family kinases. Therefore, SYK may be sufficient for ITAM-phosphorylation under certain conditions. In line with previous studies (27, 43), we detected CD3ζ phosphorylation in the presence of SYK and lower phosphorylation in the presence of ZAP70, supporting this hypothesis. ZAP70-induced SLP-76 phosphorylation was enhanced when we co-expressed Fyn, suggesting that ZAP70 is more involved in the typical ITAM signaling pathway. This may be correlated with the fact that we saw weaker CD3ζ phosphorylation induced by ZAP70 compared to SYK so that additional phosphorylation by Fyn could enhance this signaling pathway. Additionally, Fyn-mediated phosphorylation of specific tyrosine residues within the interdomain separating the C-terminal SH2 domain and the kinase domain of ZAP-70 are important for regulating the activity of ZAP-70 (16, 17).

There have been several studies demonstrating that SYK may be more important for the function of ITAM-based receptors in NK cells than ZAP70 (44–46). SYK was found to be essential for DAP12 signaling, whereas ZAP70 was dispensable (45). The dominance of SYK over ZAP70 may be influenced by the fact that SYK can engage a non-typical ITAM-based signaling pathway independent of Src-family kinases. In CD45-deficient NK cells, CD45 independent ITAM signaling is mediated by SYK, but not by ZAP70 (46). In the absence of CD45, Src-family kinases cannot be activated as the inhibitory tyrosine is not dephosphorylated, thus only the non-typical SYK activation without Src-family kinases would remain active. CD45-deficient mice have few peripheral T cells since TCR signaling during thymic development is greatly impaired (47, 48). However, B cell development is not impaired in these mice, further supporting our model that SYK and ZAP70-based ITAM signaling are differentially dependent on Src-family kinases.

In contrast to T cells, inhibition of Src-family kinases did not abolish ITAM-mediated SLP-76 and SLP-65 phosphorylation in NK cells and B cells, respectively. However, it did reduce the phosphorylation levels of SLP-76 and SLP-65. This suggests that the typical and the non-typical ITAM signaling pathways may both be operational in these cells. In B cells, the use of both pathways may be dependent on the way the BCR is engaged (8, 9, 49, 50) and the differential regulation of SYK by Src-family kinases could calibrate the threshold for BCR receptor activation. It is interesting to speculate if a similar mechanism applies to NK cells. As NK cells not only express SYK but also ZAP70, functional differences could be regulated by differential expression of both kinases. Additionally, SYK and ZAP70 can also function as adapter proteins (51, 52) and differences in interaction partners could influence specific signaling pathways initiated by SYK or ZAP70. Finally, loss of SYK expression has been associated with a memory-like phenotype of NK cells after HCMV infection (53, 54). Further studies are therefore warranted to investigate how the differential use of typical and non-typical ITAM signaling pathways influences NK cell functions.

## Author Contributions

FF and CW designed the experiments and wrote the manuscript. FF performed experiments. MC and SW conducted the cell sorting and MS performed cloning.

## Conflict of Interest Statement

The authors declare that the research was conducted in the absence of any commercial or financial relationships that could be construed as a potential conflict of interest.
