# Supplementary material for: Differential Requirements for Src-Family Kinases in SYK or ZAP70-Mediated SLP-76 Phosphorylation in Lymphocytes
Source: Front Immunol. 2017 Jul 7;8:789. doi: 10.3389/fimmu.2017.00789 (PMC5500614; doi:10.3389/fimmu.2017.00789)
Supplement: Supplementary file 1 [file data_sheet_1.pdf]

Supporting Information

Differential requirements for Src-family kinases in SYK or ZAP70 mediated SLP-76 phosphorylation in lymphocytes

Frank Fasbender, Maren Claus, Sabine Wingert, Mina Sandusky, Carsten Watzl*

Leibniz Research Centre for Working Environment and Human Factors, IfADo, TU-Dortmund, D-44139 Dortmund, Germany

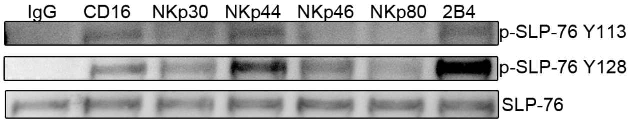

Supplementary Figure 1: SLP-76 phosphorylation in cultured human NK cells after cross-linking of activating receptors. IL-2 activated, expanded NK cells (isolated and cultured as described in the methods section) were stimulated with mAbs specific to the indicated activating NK cell receptors by cross-linking with secondary goat F(ab′)$_2$ anti-mouse IgG at 37°C for 2 min. Phosphorylation of SLP-76 was analyzed by Western Blot.

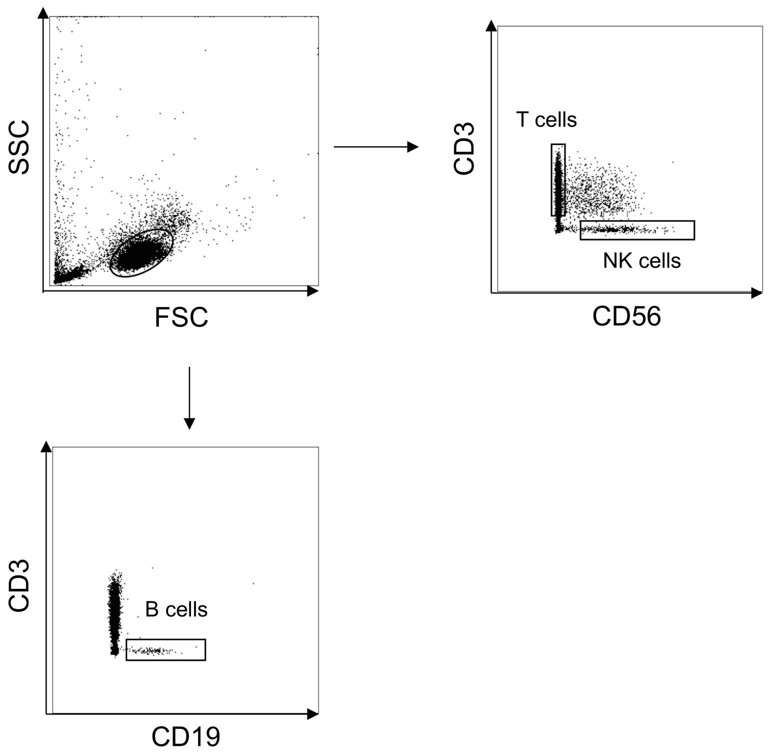

Supplementary Figure 2: Gating for Phosflow analysis. First a FSC/SSC-plot was made to gate on lymphocytes. NK cells were identified as CD3-CD56+, T cells as CD3+CD56 and B cells as CD3-CD19+.
